# Computed tomography angiography of aorta subjected to external wrapping

**DOI:** 10.1186/s13019-016-0487-y

**Published:** 2016-06-01

**Authors:** Tomasz Płonek, Andrzej Dumanski, Rafal Nowicki, Wojciech Kustrzycki

**Affiliations:** Department of Cardiac Surgery, Wroclaw Medical University, Borowska 213, 50-556 Wroclaw, Poland

## Abstract

**Background:**

External wrapping is a surgical technique used in patients with dilated ascending aorta. To date, there is no available data describing the radiographic features of the aorta subjected to external wrapping using a straight corrugated Dacron vascular prosthesis. The aim of this study was to find distinctive radiographic features of an externally constricted aorta.

**Methods:**

Preoperative and early postoperative (7th postoperative day) CT angiography images of ten patients who underwent wrapping procedures were assessed and compared. The images were analyzed in order to find characteristic features of CT angiography images of the ascending aorta subjected to external wrapping.

**Results:**

The CT-angiography images showed that the aortic wall deformed significantly (the wall plicated) after the wrapping procedure in one patient, whose aortic diameter was decreased by 47 %. The remaining nine patients did not have significant aortic wall deformations. All patients presented with a periaortic mass. This was a collection of blood clots and pericardial fluid that filled the empty space in the pericardium following a decrease in the diameter of the ascending aorta. A very thin (<1 mm) crescent-shaped uncontrasted layer was noticed between the aorta and the periaortic area in all patients. This, in turn, was an empty space between the aorta and the corrugated vascular prosthesis.

**Conclusions:**

The CT-angiography images of the aorta subjected to external wrapping may have unique features that are not observed after other operations on the ascending aorta. The knowledge about the details of this surgical procedure helps to correctly assess these images.

## Background

External wrapping is a surgical technique which consists in placing an external corset around a dilated fragment of the vessel [1]. The operation is aimed at reducing the risk of aortic complications by preventing the vessel from further dilatation. According to previously published data, aortic wrapping restores the normal diameter of the aorta, is characterized by low mortality and prevents the aorta from further dilatation [2].

Several materials are used for the wrapping procedure. Some surgeons use a straight corrugated Dacron vascular prosthesis but the procedure can also be done using the Dacron mesh or cellophane [3–9]. One technique is to “wrap” the aorta without decreasing its diameter. Another approach aims to reduce its diameter [3, 8–12].

The procedure is usually performed in patients with a moderately dilated ascending aorta as a concomitant procedure to other cardiac surgery operations, i.e. aortic valve replacement. Sometimes it is performed as an isolated procedure [2, 8, 9]. It can also be performed in patients with the Marfan syndrome [13]. The latter procedure is called the PEARS technique (Personalized External Aortic Root Support) and differs significantly from the classic wrapping procedure as the geometry of the wrap is prepared before the operation according to the three dimensional reconstruction of the imaging studies of a specific patient.

As the aorta is subjected to the external wrapping, i.e. using the off-the-shelf corrugated vascular tube graft, its shape changes, especially if its diameter is markedly reduced. What is more, this technique may lead to a deformation and plication of the aortic wall. There are no studies reporting radiographic characteristics of a surgically constricted ascending aorta with the use of external wrapping. One way to establish the standard angio-CT image of the wrapped aorta is an analysis of the early postoperative angio-CT images of patients who underwent this procedure.

The aim of this study was to compare the preoperative and early postoperative angio-CT images of patients with a dilated aorta undergoing external wrapping of the ascending aorta using the corrugated Dacron vascular tube graft and to find distinctive features of an externally constricted aorta.

## Methods

Preoperative and early postoperative (7th postoperative day) CT angiography images of ten patients who underwent a wrapping procedure were analyzed. All the CT angiograms were ECG-gated with a 0.625 mm slice thickness. The CT angiograms were evaluated by two observers experienced in analyzing cardiovascular CT scans. The true cross-section of the most dilated part of the tubular aorta was obtained in all patients by aligning the image so that the measurements could be taken in a plane perpendicular to the long axis of the aorta.

**Fig. 1:**
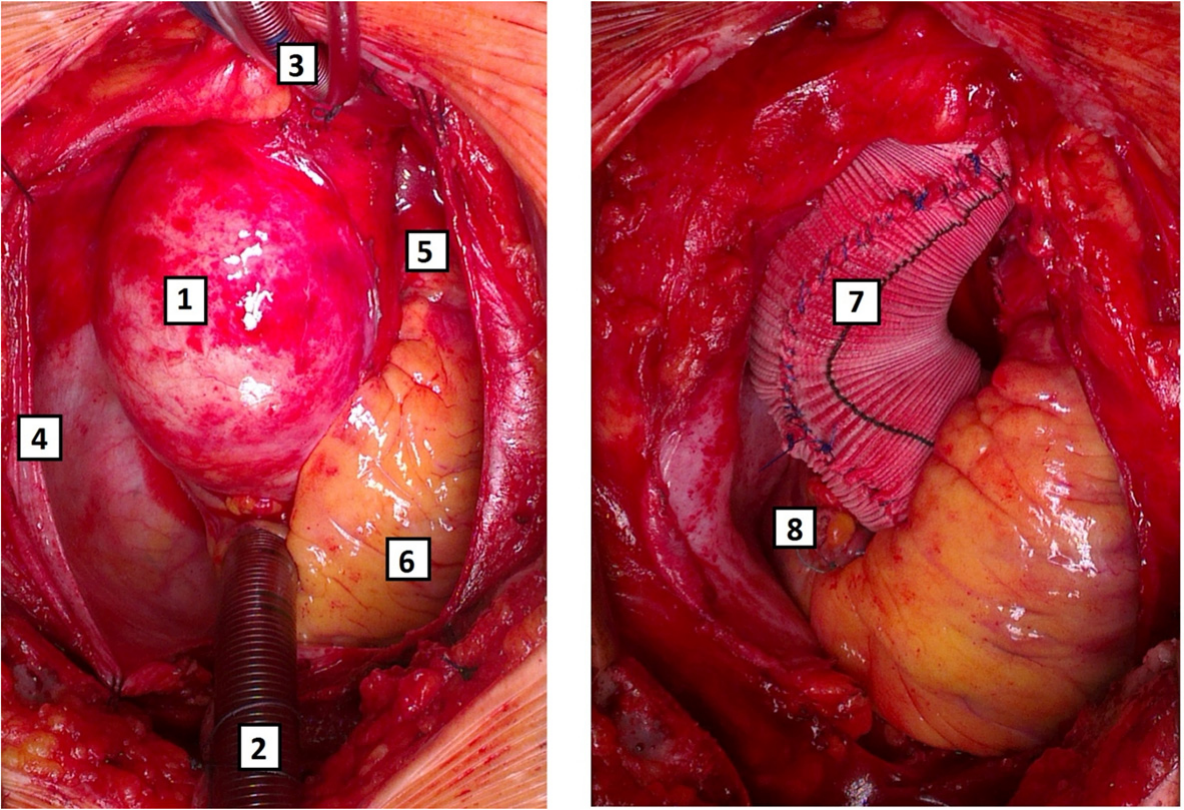
Preoperative (left) and postoperative (right) photos of the aorta being wrapped with a Dacron vascular prosthesis. 1 – aorta, 2 – venous cannula for the heart-lung machine placed in the right atrial appendage, 3 – arterial cannula for the heart-lung machine placed in the proximal aortic arch, 4 – pericardial sac, 5 – pulmonary trunk, 6 – right ventricle, 7 – wrapped aorta, 8 – right atrium

During the surgical procedure, the dilated tubular part of the ascending aorta was wrapped using a 34–36 mm wide off-the-shelf corrugated straight Dacron vascular tube graft. The surgery was performed through a standard median sternotomy with each patient being connected to the heart-lung machine. The vascular prosthesis was cut longitudinally and placed around the dilated segment of the aorta (from the sinotubular junction to the innominate artery). Subsequently, the edges of the prosthesis were approximated and stitched using a 3-0 nonabsorbable monofilament suture. The proximal and distal ends of the prostheses were sutured to the surface (adventitia) of the aorta using several 5-0 nonabsorbable monofilament stitches to prevent dislocation of the wrap (Fig. 1). The aortic wall was left intact in all patients. Most patients (*n* = 8, 80 %) underwent concomitant aortic valve replacement. Two patients underwent concomitant aortic valve repair. The mean age of the patients was 72.8 ± 6.8 years and 7 (70 %) of the patients were males.

The preoperative aortic diameters were compared to the postoperative ones. The images were analyzed in order to find potential sites of deformation of the aortic wall as well as other characteristic radiographic features of an external wrapping of the ascending aorta.

## Results

### Aortic diameter

**Fig. 2:**
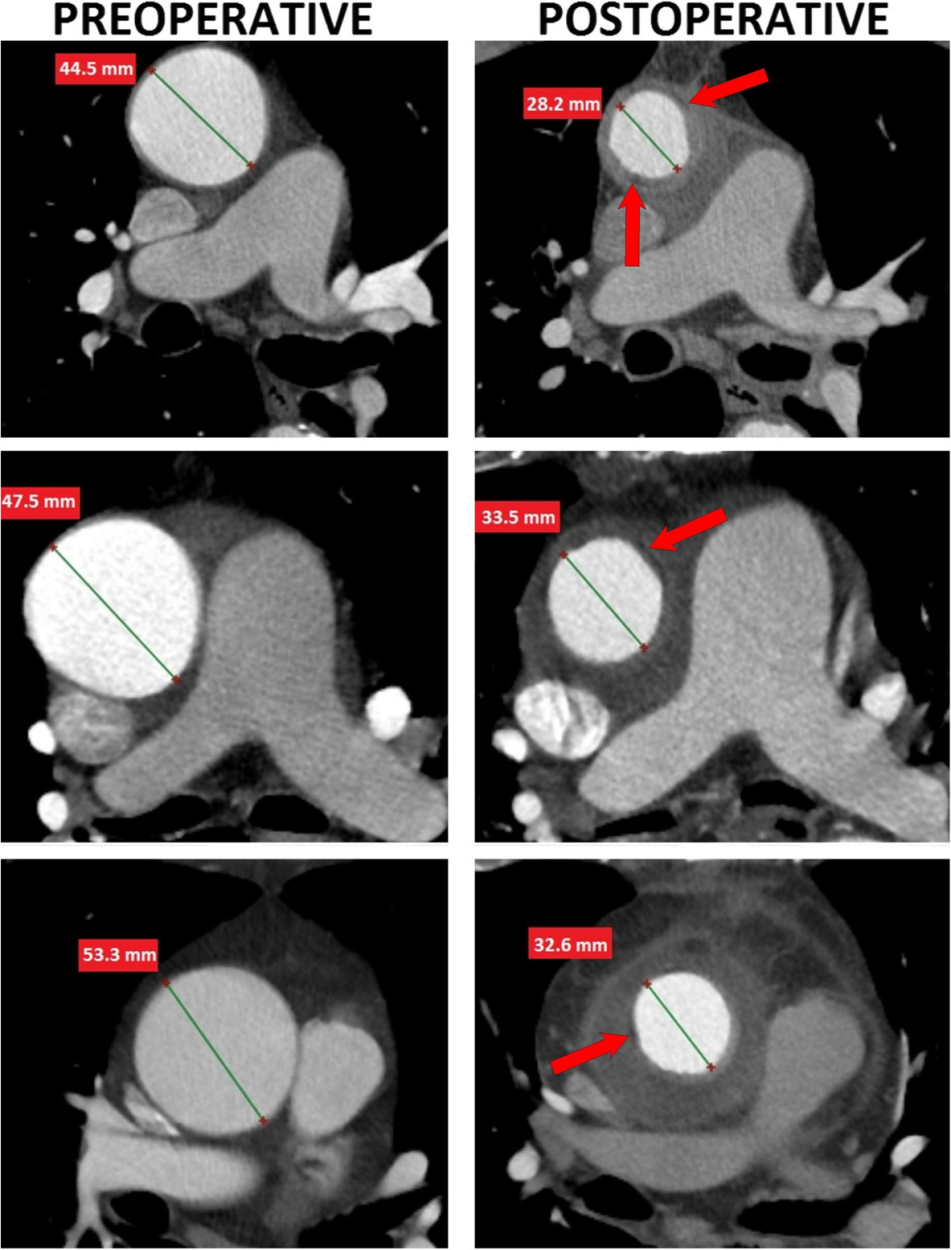
Preoperative and postoperative angio-CT images of patients who underwent the wrapping procedures. Red arrows point toward the uncontrasted layer between the vascular prosthesis and the aortic wall

**Fig. 3:**
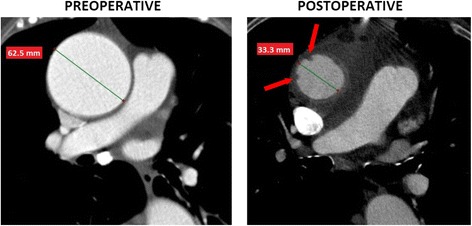
A comparison of preoperative and postoperative angio-CT images of a patient whose aorta was decreased by 47 %. Red arrows point at the sites of plication of the aortic wall

The largest mean preoperative diameter of the ascending aorta was 50.5 ± 4.8 mm (range: 45–62.5 mm) and was reduced on average by 39 % (range: 36 %–47 %) following the wrapping procedure. The largest mean postoperative aortic diameter was 30.7 ± 1.5 mm (range:29–33.3 mm). The postoperative diameter of the wrapped portion of the aorta was on average 4.5 ± 0.9 mm smaller than the Dacron vascular prosthesis used for the procedure. The comparison of the preoperative and postoperative angio-CT images of the aorta is presented in Figs. 2 and 3.

### Aortic wall

No intimal flap or double lumen, suggesting the presence of aortic dissection, were found in the early postoperative CT angiography images. Aortic wall plication was present in one patient, whose aortic diameter was decreased by 47 % (from 62.5 to 33.3 mm). There were two filling defects in the aortic lumen located one cm above the sinotubular junction. One of these was 4mm×3.5 mm large and the other measured 3mm×1.5 mm (Fig. 3). There were no visible plications of the aortic wall in the remaining patients. The aortic lumen preserved a circular shape postoperatively. However, the inner surface of the aortic wall seemed not to be completely smooth.

### Periaortic area

The space in the mediastinum vacated after the reduction of the diameter of the aorta was filled with postoperative blood clots and pericardial fluid. Therefore, the postoperative angio-CT images revealed a cuff of low attenuation in all patients (Fig. 2). Moreover, a very thin (<1 mm) crescent-shaped uncontrasted layer between the aorta and the periaortic area was found in all patients, which corresponded to empty space between the aorta and the convex part of the corrugation of the vascular prosthesis.

## Discussion

Patients with aortic valve pathology requiring surgical treatment often have moderately dilated aortas. So far, there is no consensus whether a moderately dilated aorta should be replaced during aortic valve surgery or not. A recently published study suggests that in most cases, the aorta dissects before reaching a diameter which is a threshold for standard surgical correction (a replacement of the ascending aorta) [14, 15]. Therefore, patients with moderately dilated aortas may benefit from operations aimed at reinforcing their aorta and protecting it from further dilatation, i.e. aortic wrapping. The surgical outcomes of patients treated using this technique are promising [2, 3, 8, 9, 11]. The aorta is not excised during this procedure, its diameter is decreased and it receives an additional scaffold made of a vascular prosthesis.

Patients undergoing surgeries for aortic aneurysms are followed-up during the early postoperative period to exclude serious iatrogenic postoperative aortic complications, i.e. aortic dissection. We determined some characteristic radiographic features that may be found in CT angiography of patients after external wrapping in which the aortic diameter is reduced.

A very thin, crescent-shaped uncontrasted periaortic layer, which was an empty space between the constricted aorta and the corrugated vascular prosthesis, was observed in all patients in this study. The diameter of the aorta was significantly decreased during the procedure. Patients whose aortic diameter was decreased by less than 40 % did not have any visible aortic wall plications. A plication of the aortic wall was found in only one patient whose aortic diameter was decreased by almost 50 %. This finding suggests that external wrapping should not be routinely performed in patients whose aorta is dilated above >60 mm, as it has less elasticity, causing the redundant aortic wall to plicate. However, there are no data clarifying whether a plicated aortic wall is more prone to degeneration and dissection. There have been reports of complications following external wrapping of the aorta, although these were associated with the dislocation of the corset and subsequent aortic redilatation rather than aortic wall plication [16, 17]. One of the explanations why the wall of a moderately dilated aorta does not plicate following a reduction in its diameter is that it still possesses some elasticity and the blood pressure which pushes the wall against the vascular prosthesis prevents it from plicating.

The empty space in the pericardial sac surrounding the aorta left after decreasing the aortic diameter is filled with blood clots and pericardial fluid. In some patients, this intrapericardial hematoma may resemble an intramural hematoma (IMH). However, an intramural hematoma cannot be located outside the corset made of the vascular prosthesis. Therefore, this aortic complication may also be excluded when the internal diameter of the periaortic hematoma/cuff is larger than the diameter of the vascular prosthesis used for wrapping.

External wrapping may cause the rarefaction of the aortic wall [18]. The vessel’s wall subjected to external compression may degenerate. However, there is no evidence whether this phenomenon is of clinical relevance. A recent study proves that external scaffold placed on the aorta does not increase the stress in its wall [19]. It means that the aorta, although thinned, may not be more prone to dissection. A recent study proved that the Dacron mesh does not cause aortic wall rarefaction and gets incorporated in the aortic wall, which means that this fabric may be a better option for patients undergoing wrapping procedure [20].

## Conclusions

The CT-angiography images of the aorta subjected to external wrapping may have unique features that are not observed after other operations on the ascending aorta. The knowledge about the details of this surgical procedure helps to correctly assess these images.
